# Role of *RIM101* for Sporulation at Alkaline pH in *Ashbya gossypii*

**DOI:** 10.3390/jof7070527

**Published:** 2021-06-30

**Authors:** Lisa Wasserstrom, Jürgen Wendland

**Affiliations:** 1 Carlsberg Research Laboratory, Carlsberg A/S, 1799 Copenhagen V, Denmark; surfabettan@gmail.com; 2 Department of Microbiology and Biochemistry, Hochschule Geisenheim University, Von-Lade-Strasse 1, 65366 Geisenheim, Germany

**Keywords:** filamentous fungus, signal transduction, meiosis, germination, ascus, functional analysis

## Abstract

Microorganisms need to sense and adapt to fluctuations in the environmental pH. In fungal species, this response is mediated by the conserved *pacC*/*RIM101* pathway. In *Aspergillus nidulans*, PacC activates alkaline-expressed genes and represses acid-controlled genes in response to alkaline pH and has important functions in regulating growth and conidia formation. In *Saccharomyces cerevisiae*, the PacC homolog Rim101 is required for adaptation to extracellular pH and to regulate transcription of *IME1*, the Initiator of MEiosis. *S. cerevisiae rim101* mutants are defective in sporulation. In *Ashbya gossypii*, a filamentous fungus belonging to the family of *Saccharomycetaceae*, little is known about the role of pH in regulating growth and sporulation. Here, we deleted the *AgRIM101* homolog (AFR190C). Our analyses show that Rim101 is important for growth and essential for sporulation at alkaline pH in *A. gossypii*. Acidic liquid sporulation media were alkalinized by sporulating strains, while the high pH of alkaline media (starting pH = 8.6) was reduced to a pH ~ 7.5 by these strains. However, *Agrim101* mutants were unable to sporulate in alkaline media and failed to reduce the initial high pH, while they were capable of sporulation in acidic liquid media in which they increased the pH like the wild type.

## 1. Introduction

All microorganisms need to respond to environmental changes in order to survive. This often involves signal transduction pathways that transcriptionally induce genes to improve the species’ adaptation. In many fungal species, the response to changes in extracellular pH is mediated by the conserved *pacC/RIM101* pathway [1]. 

Gene regulation by ambient pH has been most extensively studied in *Aspergillus nidulans*, where it is mediated by the C2H2 zinc finger transcription factor PacC. In response to alkaline pH, PacC acts both as an activator and a repressor by binding the consensus sequence GCCARG. Alkaline-expressed genes are activated while acid-controlled genes are repressed [2]. PacC plays an essential role in pulmonary pathogenesis in *A. nidulans* [3] and deletion results in poor growth and conidiation [4]. Orthologs of *pacC* are also important for virulence in other fungal species. 

In the human pathogen *Candida albicans*, Rim101 is essential for dimorphism and virulence since it regulates alkaline pH-induced filamentation [5,6]. In the plant pathogen *Fusarium oxysporum*, PacC most likely acts as a negative regulator of virulence by inhibiting transcription of acid-expressed genes that are essential for pathogenicity [7]. In *Magnaporthe oryzae* deletion of PacC abolishes conidiation at pH 8 but only reduces virulence [8]. 

**Figure 1:**
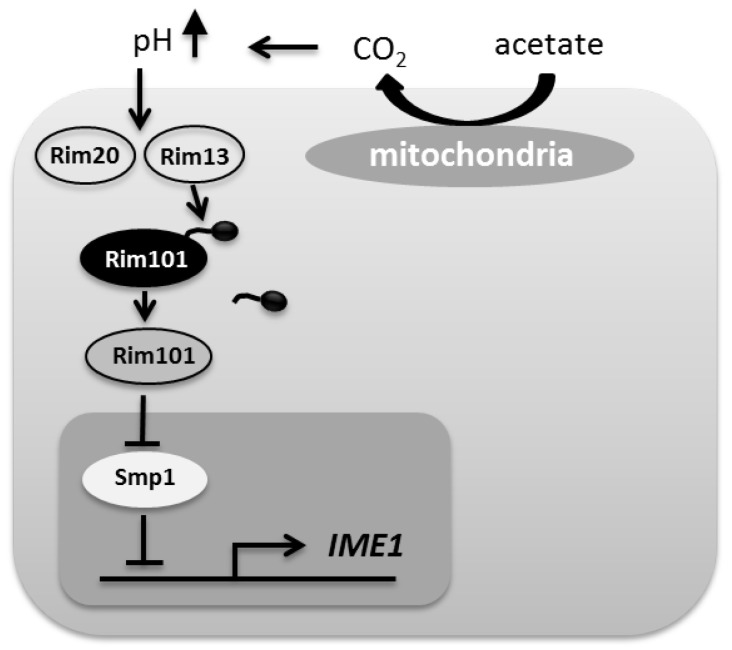
Induction of sporulation in response to alkaline pH in *S. cerevisiae*. When a non-fermentable carbon source is metabolized by respiration the external pH increases. The alkaline pH induces proteolytic activation of Rim101 by Rim13/Rim20. Rim101 then activates *IME1* transcription by repressing transcription of the repressor Smp1 [14].

In *Saccharomyces cerevisiae*, the PacC homolog ScRim101 is a transcriptional repressor that is required for adaptation to extracellular alkalization by regulating expression of genes encoding ions pumps and transporters [9]. In contrast to PacC in *A. nidulans*, which acts directly as a transcriptional activator, Rim101 in *S. cerevisiae* activates genes indirectly via repressing transcriptional repressors, mainly *NRG1* and *SMP1*. ScRim101-mediated repression of *SMP1* transcription results in activation of *IME1* expression [10]. A prerequisite for *IME1* activation by ScRim101 is the absence of glucose and presence of non-fermentable sugars, which, when metabolized, leads to an alkalization of the media. This triggers the highly conserved alkaline-sensing pathway to activate Rim101 by proteolytic cleavage catalyzed by the Rim13 protease (Figure 1) [11,12,13]. In *A. nidulans*, this proteolytic cleavage is mediated by the signaling protease PalB [12]. Deletion of *ScRIM101* results in a strain severely reduced in sporulation that fails to induce haploid-invasive growth in response to alkaline pH. A *Scrim101* mutant grows poorly at alkaline pH and low temperature and is sensitive to Na^+^ and Li^+^-ions [9].

The filamentous ascomycete *Ashbya gossypii* is a pre-whole genome duplication fungus that belongs to the family of the yeasts *Saccharomycetaceae* and shares homologs of 95% of its genes with *S. cerevisiae* [15]. Previous studies have shown that the main regulators of sporulation in *S. cerevisiae* are also essential for sporulation in *A. gossypii*, including *IME1*, *IME2*, *IME4*, *KAR4*, *NDT80*, and also the cAMP/Protein Kinase A pathway [16,17,18]. In *S. cerevisiae*, *IME1* expression requires ScRim101, and sporulation is therefore dependent on alkaline pH [19]. In this study, we investigated the role of pH on sporulation and vegetative growth in *A. gossypii* by deleting the *AgRIM101* homolog. Our results show that *AgRIM101* is important for both sporulation and growth only at alkaline pH in *A. gossypii*. However, *RIM101*-mediated signaling at alkaline pH is not a prerequisite for sporulation in *A. gossypii,* since both the wild-type and the *rim101* mutant strains were able to sporulate abundantly when incubated at acidic starting pH (pH 5.3), which increased to ~ pH 7.2 due to metabolic activity.

## 2. Materials and Methods

### 2.1. Strains and Media

*A. gossypii* strains were grown in *Ashbya* Full Medium (AFM) (1% yeast extract, 1% peptone, 2% dextrose), and G418/geneticin (200 μg/mL) was used for selection of antibiotic-resistant transformants. For pH studies, full media plates were buffered with Tris-HCl to pH 6.5, 7.0, 7.5, 8.0, and 8.5. In sporulation assays, full media plates supplemented with 1 g/L myo-inositol were used. Liquid sporulation assays were carried out in a minimal medium (1.7 g/L yeast nitrogen base (YNB), w/o ammonium sulfate and w/o amino acids, 0.69 g/L CSM (Formedium, Hunstanton, UK), 20 g/L glucose, 2 g/L asparagine, and 1 g/L myo-inositol) buffered with Tris-HCl to pH 8.5 where indicated. Two independent transformants were generated for the *Agrim101* deletion strain. The strains used in this study are listed in Table 1.

### 2.2. Transformation of A. gossypii 

*A. gossypii* was transformed by electroporation as described previously [20]. G418/geneticin (200 μg/mL) was used for selection of antibiotic-resistant transformants. The *A. gossypii rim101* deletion strains were generated by amplifying PCR cassettes from pFA-GEN3 using gene-specific S1- and S2-primers. These PCR cassettes were then used as transforming DNA [21]. All oligonucleotide primers were purchased from LGC genomics GmbH (Germany) and are listed in Table 2. To verify the correct integration of the cassettes and absence of the target gene in two independent homokaryotic deletion strains, diagnostic PCR was used. 

### 2.3. Sporulation Assay

The sporulation ability at different pH values was determined in the wild-type strain and the *Agrim101* mutant by isolating spores from the central part of colonies grown for 10 days at 30 °C on full media plates buffered with Tris-HCl to pH 6.5, 7.0, 7.5, 8.0, and 8.5. A circle of the central mycelia (15 mm in diameter) was cut out from the plate and suspended in a 5 mL TE buffer containing 200 µL zymolyase (10 mg/mL) to degrade vegetative hyphal cells and release the spores. After 3 hours of incubation at 37 °C (on a tilting rotor) the spores were collected by centrifugation and washed twice in spore buffer (0.03 % Triton-X-100). Serial dilutions were performed in spore buffer and 100 µL of the appropriate dilutions were plated on full media plates and incubated at 30 °C until colonies appeared. It should be noted that single-spore suspensions are difficult to obtain with *Ashbya* due to the hydrophobicity of the spores and a terminal filament at the proximal end of each spore, which leads to the agglomeration of spores. The CFUs therefore derive mostly from clumps of spores. The data are, thus, presented as the number of colony forming units/mL.

Sporulation in liquid culture was monitored by transferring mycelia pre-cultured in AFM to a minimal medium. These cultures were then incubated for one week at 28 °C. Thereafter, sporulation was observed by microscopy and end-point pH was measured. Experiments were done three times and representative data are shown. Clumps of spores were easily identified by microscopy. Under these conditions *Ashbya* mycelia sporulate quantitatively. Alkaline pH was obtained by buffering minimal media (pH 5.3) with Tris-HCl to pH 8.6.

### 2.4. Microscopy

Microscopic images of sporulating cultures were observed using a fully motorized Zeiss Axiovert 200M microscope equipped with a Plan-Neofluar 100×/1.30 objective (Carl Zeiss AG, Feldbach, CH). Imaging was controlled by AxioVision 4 software and single-plane brightfield images were acquired using an AxioCam MRm Rev.3 and compiled using Fiji/ImageJ 2.1.0/1.53c.

## 3. Results

### 3.1. Rim101 Has No Role in Vegetative Growth in A. gossypii

**Figure 2:**
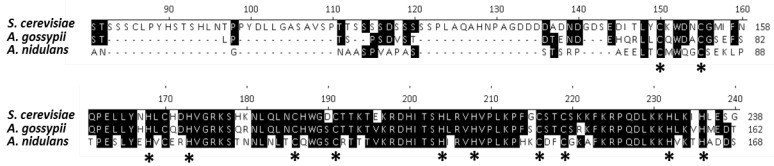
Alignment of the zinc finger regions Rim101/PacC proteins of *S. cerevisiae*, *A. gossypii*, and *Aspergillus nidulans*. The conserved cysteine and histidine residues of the three C2H2 zinc-chelating residues are marked with asterisks [4].

*Ashbya RIM101* has been identified in the *Ashbya* Genome Database and annotated as *AFR190C* being a syntenic homolog of the *S. cerevisiae YHL027W* [22]. The conserved C2H2 zinc finger domains are shown in Figure 2. Similarly, *RIM13*/*YMR154C* and *RIM20*/*YOR275C* are also highly conserved in *Ashbya* as *AgRIM13*/*ADR274C* and *AgRIM20*/*AER342C*.

Using the well-established and highly efficient PCR-based gene-targeting approach, we generated independent *rim101* deletion strains in *A. gossypii* that were verified by standard diagnostic PCR [20,21]. Gene deletions generate heterokaryotic mutants that harbor both wild-type and transformed nuclei in their hyphal compartments. Usually, and also in our study, heterokaryotic transformants harbor wild-type phenotypes. Sporulation of these heterokaryotic strains allows for the selection of uninucleate mutant spores based on G418 antibiotic selection, which germinate and generate homokaryotic mycelia harboring only nuclei with the linked mutation. Two independent mutant strains were generated that showed similar phenotypes. Both mutants were analyzed in parallel and representative images of one strain (AWL63) are shown unless otherwise indicated (see Table 1).

**Figure 3:**
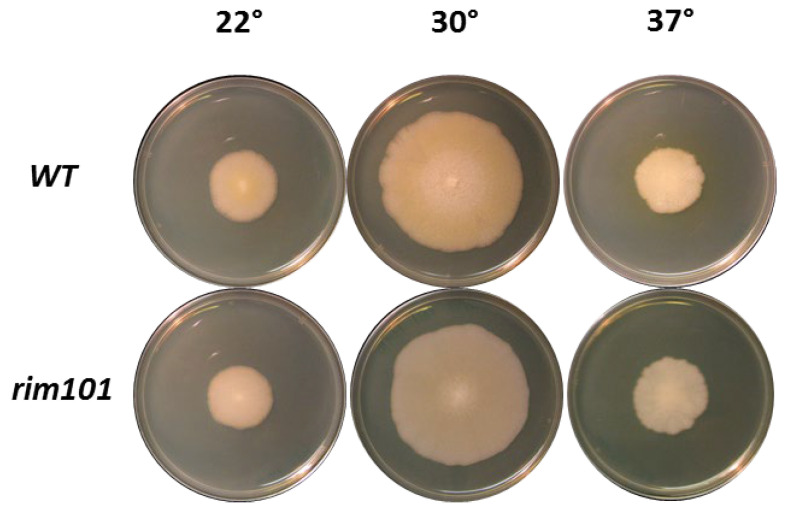
Radial growth of the *Ashbya* parental (WT) strain and the *Agrim101* mutant (AWL63) on full medium plates after one week of growth at the indicated temperatures.

The effects on vegetative growth in the *Agrim101* mutant were studied comparatively by plating strains on full medium plates and monitoring radial growth for 7 days at various temperatures (22 °C, 30 °C, and 37 °C). Under these conditions, deletion of *RIM101* in *A. gossypii* did not reveal a negative effect on vegetative growth compared to the wild type (Figure 3). 

### 3.2. Sporulation at High pH in A. gossypii Requires Rim101

**Figure 4:**
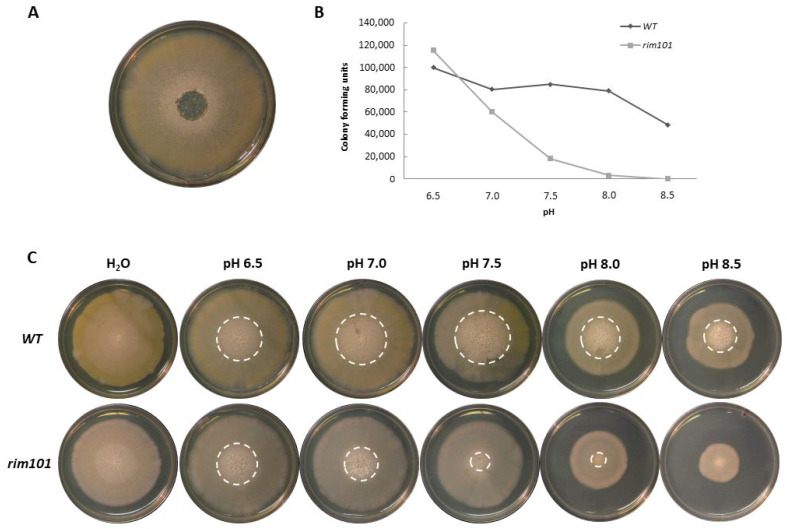
Sporulation ability of the *A. gossypii* wild-type strain and *rim101* mutant at different pH. (**A**) Spores were isolated from the central part of colonies grown for 10 days on a full medium plate. (**B**,**C**) Sporulation of mycelia on solid media buffered with Tris-HCl to different pH values. (**B**) Spore-forming units were determined by dilution plating of spore suspensions derived from the sporulation zones. (**C**) Sporulation zones of mycelia were marked with white-dotted circles.

Next, we went on to investigate the role of *AgRIM101* for growth under alkaline pH conditions. We monitored radial growth of the wild-type and the *Agrim101* mutant on full medium plates set to pH 6.5, 7.0, 7.5, 8.0, and 8.5. This indicated reduced vegetative growth of the *Agrim101* mutant at the alkaline pH 8.0–8.5 (Figure 4).

Sporulation in *S. cerevisiae* is only initiated at alkaline pH when Rim101 is functional and promotes *IME1* expression via repression of *SMP1*. Deletion of *RIM101* in yeast, therefore, results in a generally sporulation-deficient strain [23]. Thus, we studied the effect of pH on sporulation in the *A. gossypii* wild-type and *Agrim101* mutant strains. Sporulation commences from central parts of *Ashbya* mycelia and leads to sporangium formation of cell compartments and mycelial fragmentation. We isolated spores from these central parts of mycelia of strains grown for 10 days on full media plates buffered to different pH levels (as shown in Figure 4A). Dilutions of the spore suspensions were plated and the numbers of colony-forming units were determined (Figure 4B). This showed that *AgRIM101* is required for sporulation at alkaline pH since sporulation efficiency dropped several orders of magnitude in the *Agrim101* mutant at pH 8.0 and 8.5. However, both the *Ashbya* wild-type strain and the *Agrim101* strain sporulated equally well at pH 6.5. This indicates a differential response to pH in *A. gossypii* resulting in the inability to sporulate when challenged by high pH. Both the *Ashbya* wild-type strain and the *Agrim101* mutant formed similar-sized sporulation zones at pH 6.5. However, while the sporulation zone in the wild-type strain increased at pH 7.0 and 7.5, this zone got smaller and smaller in the *rim101* mutant challenged by higher initial pH values. 

**Figure 5:**
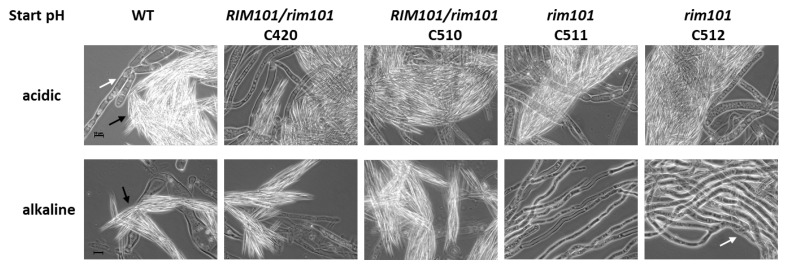
Liquid sporulation assays at acidic and alkaline pH. The indicated strains were pre-grown in AFM and then transferred to CSM-sporulation media (acidic = unbuffered with a starting pH of 5.3; alkaline = Tris-buffered to a pH of 8.6 at room temperature). After one week of incubation at 28 °C, samples were analyzed by microscopy. Representative images are shown. Clumps of spores (indicated by black arrows in the WT) were abundant in sporulating strains but completely absent in homokaryotic *rim101* mutants showing only hyphal filaments (indicated by white arrows). Scale bars are 10 µm.

Radial colony growth at alkaline pH was reduced in the *rim101* mutant compared to the wild type. Accordingly, the sporulation zone may have been decreased promoting the finding of lack of sporulation under these conditions. To analyze this in more detail we investigated sporulation potential at different pH values in liquid media. *A. gossypii* is able to sporulate abundantly in liquid media. Thus, the wild-type, heterokaryotic, and homokaryotic *rim101* strains where pre-grown overnight in AFM and equal amounts of mycelia were transferred to the minimal media of acidic (pH 5.3) and Tris-buffered alkaline (pH 8.6) starting pH values. After one week of incubation, the cultures were analyzed. The wild-type and heterokaryotic mutants sporulated profusely under both conditions (Figure 5).

Sporulation of *rim101* mutants, however, was specifically blocked in the alkaline medium, while they sporulated well in the acidic medium. Sporulation of *rim101* mycelia was as abundant as in the wild-type strain under acidic starting conditions, while it was completely abolished in liquid cultures with an alkaline starting pH, where no spores were found at all. This indicates that an alkaline response via *RIM101* is required to enable sporulation in *Ashbya* at elevated pH.

Cellular metabolism and adaptation to growth media results in alterations in media pH. Therefore, we determined the endpoint pH of these cultures. The pH of the acidic minimal media was raised to between pH 6.7 and pH 7.6 (WT pH 7.6; heterokaroytic strains C420 pH 6.7 and C510 pH 7.1; homokaryotic strains C511 pH 7.2 and C512 pH 7.0). The pH of the alkaline-buffered media was lowered in the sporulating strains of the wild type to pH 7.8 and in the heterokaryotic strains to pH 7.0 (C420) and pH 7.2 (C510). The homokaryotic *rim101* mutant strains, however, failed to substantially reduce this alkaline pH, which remained high at pH 8.4 for both strains. Both homokaryotic *rim101* strains failed to sporulate under these alkaline pH conditions (Figure 5).

## 4. Discussion

Fungi are able to grow over a wide range of extracellular pH and must therefore be able to sense and respond to changes in environmental pH. In fungi, this is mediated by the conserved Rim101/PacC pathway. In both *S. cerevisiae* and *A. nidulans* Rim101 and PacC are important developmental regulators as they promote sporulation and conidiation in these fungi, respectively. In alkaline media, *A. nidulans* PacC activates expression of alkaline genes and represses acidic-expressed genes, while this does not occur in acidic conditions [1 and references therein]. This is different in *S. cerevisiae*, where Rim101 acts indirectly by repressing the regulators *NRG1* and *SMP1* [10].

In our study, we showed that in *Ashbya*, the Rim101 homolog has a role in in adaptation to alkaline pH and sporulation under elevated pH conditions.

Our functional analysis of *AgRIM101* shows that there are several differences to *ScRIM101*. *AgRIM101* is not required for vegetative growth at 22 °C, 30 °C, and 37 °C, which is in contrast to *S. cerevisiae*, as *Scrim101* mutants grow poorly at low temperatures [24]. Previously, it was shown in *S. cerevisiae* that Rim101 is required for a proper response to salt stress [25]. When the *A. gossypii rim101* mutant was subjected to NaCl salt stress only a slightly increased sensitivity to 0.4 M NaCl was observed, resulting in uneven radial growth compared to the wild type.

Loss of AgRim101, however, was found to reduce radial growth at pH 7.5–8.5, and we also observed increased colony lysis of older mycelia. This phenotype is somewhat similar to that observed in the *Agmkk1* and *Agbck1* cell wall integrity mutants [26]. This suggests a link in *Ashbya* between the RIM and PKC pathways in cell wall maintenance as was shown for *S. cerevisiae*, where *RIM101* was found to be synthetic lethal with the cell wall integrity MAP Kinase *SLT2* [27].

AgRim101 is not required for sporulation at an acidic starting pH since the *Agrim101* mutant sporulates at approximately wild-type levels under standard laboratory sporulation-inducing conditions. Under these conditions, the metabolism of both wild-type and mutant strains achieves alkalization of the medium to slightly above pH 7.0. However, *Agrim101* mutants fail to sporulate in alkaline media. Under these conditions, *RIM101* mycelia are able to reduce the pH from alkaline towards neutral pH. In these aspects, *Ashbya* differs from the sporulation-deficient phenotypes seen in both *S. cerevisiae* and *A. nidulans rim101* and *pacC* mutants [28]. 

Despite its redundancy at acidic/neutral pH, Rim101 clearly also regulates the adaptation at alkaline pH in *A. gossypii*, since both vegetative growth and sporulation are affected at pH 7.5–8.5 in the *Agrim101* mutant. 

In *Ashbya*, Rim101 may act as a repressor of sporulation-specific genes. However, in that case, more abundant sporulation would be expected in the *Agrim101* mutant at least under acidic conditions. Such an oversporulation phenotype was observed, e.g., in *Agste12* mutants but not in *Agrim101* [15]. It is not known if AgRim101 shares the same DNA-binding site as ScRim101. If so, there is a potential Rim101-binding site in the *IME1* promoter with the motif GCCAAG present 517 bp upstream of the start codon. This needs to be studied in more detail. In a promoter deletion analysis of *AgIME1* we showed, however, that 491 bp of the intergenic region upstream of *IME1* are sufficient for the wild-type-like transcriptional activation of *IME1* [29]. With this, we can conclude that the presumptive Rim101-binding site at position -517 is not required for *IME1* regulation since (i) the core promoter did not result in oversporulation and (ii) sporulation at acidic/neutral pH is not affected in *Agrim101*. RNAseq transcriptomics of non-sporulating vs. sporulating mycelia showed no differential regulation of *RIM101* expression in *Ashbya* [29]. This suggests that activation of Rim101 requires pH-mediated processing similar to what has been described in *S. cerevisiae*. 

## 5. Conclusions

This study shows that in *A. gossypii* the RIM pathway does not exert a dominant role in sporulation as described in *S. cerevisiae*. This and our previous findings indicate that sporulation in *A. gossypii* is mainly controlled by nutrients such as glucose and nitrogen. Extracellular signals including pH or pheromone signaling are not required for *Ashbya* to produce spores. *A. gossypii* is able to complete its life cycle, from a haploid spore that matures into a sporulating mycelium, without the need for a mating partner. This specialized lifestyle of *A. gossypii* with a close association to insect vectors may have put strong selection pressure on evolving a developmental program that allows for rapid sporulation based on limited environmental inputs. 

## Figures and Tables

**Table 1:** Strains used in this study. *A*. *gossypii* strains used in this study. All strains are derivatives of *Agleu2*.

No	Strain	Genotype	Source
71	ATCC10895	*Agleu2*	Lab collection
C420	AWL13	*leu2*, *rim101:: GEN3/RIM101 heterokaryon*	This study
C510	AWL62	*leu2*, *rim101:: GEN3/RIM101 heterokaryon*	This study
C511	AWL63	*leu2*, *rim101:: GEN3 homokaryon derived from AWL62*	This study
C512	AWL64	*leu2*, *rim101:: GEN3 homokaryon derived from C420*	This study

**Table 2:** Oligonucleotide primers used in this study.

Oligonucleotide	Sequence 5′-to-3′ *
#1214-G2	GGGTAATTTGTCGCGGTCTGGG
#1215-G3	GCCCATCAGATTGATGTCCTCC
#4603-G1-RIM101	GCTGCTATCGGACGCAGC
#4604-G4-RIM101	GTGGAGTGCGACTTCTCC
#4605-S1-RIM101	ACGATTATACGGCCAGCCAAATAGCAAGCGCGTTACTGCATGAACgaagcttcgtacgctgcaggtc
#4606-S2-RIM101	ATTATGTTCACAGTCTGGAGTTCTGCACGGAGGGCATCAGTGTCGctgatatcatcgatgaattcgag
#4607-I1-RIM101	CACTTATGCCAGGATCACG
#4608-I2-RIM101	GCTCCCGGAGAGGTCACC

* Upper-case sequences correspond to *A. gossypii* DNA sequences and lower-case sequences correspond to annealing regions on pFA cassettes. All sequences are written from 5′ to 3′.

## Data Availability

Not applicable.
